# 

**Published:** 1911-02

**Authors:** 


					﻿Vol. XXVI.
FEBRUARY, 1913.
No. 8.
TfflE X AIS
MEDICAL
JOURNAL
“Red Back”
PUBLISHED AT AUSTIN, TEXAS. BY
F. E. DANIEL, M. D., - - - Editor an/ Proprietor^
( liMtaw
PRINTED BY THE VON BOECKMANN-JONESWO. A fl n
Subscription, $1.00 a Year, in Advance. -	-	-	- Single Copies, 10 Cents
Entered at the Postoffice at Austin, Texas, as second class mail matter.
				

